# A typology of integrated care policies in the care home sector: A policy document analysis

**DOI:** 10.3389/fpubh.2023.943351

**Published:** 2023-02-21

**Authors:** Glenn Simpson, Charlotte Entwistle, Andrea D. Short, Marcello Morciano, Jonathan Stokes

**Affiliations:** ^1^Primary Care Research Centre, University of Southampton, Southampton, United Kingdom; ^2^Department of Psychology, Lancaster University, Lancaster, United Kingdom; ^3^Centre for Primary Care and Health Services Research, University of Manchester, Manchester, United Kingdom; ^4^Marco Biagi Department of Economics, University of Modena and Reggio Emilia, Modena, Italy; ^5^Research Centre for the Analysis of Public Policies, University of Modena and Reggio Emilia, Modena, Italy; ^6^MRC/CSO Social and Public Health Sciences Unit, University of Glasgow, Glasgow, United Kingdom

**Keywords:** health policy, integrated care, care homes, long-term care, social care

## Abstract

**Background:**

Health and social care systems in many countries have begun to trial and adopt “integrated” approaches. Yet, the significant role care homes play within the health and social care system is often understated. A key first step to identifying the care home integration interventions that are most (cost-)effective is the ability to precisely identify and record what has been implemented, where, and when—a “policy map.”

**Methods:**

To address gaps relating to the identification and recording of (cost-)effective integrated care home interventions, we developed a new typology tool. We conducted a policy mapping exercise in a devolved region of England—Greater Manchester (GM). Specifically, we carried out systematic policy documentary searches and extracted a range of qualitative data relating to integrated health and social care initiatives in the GM region for care homes. The data were then classified according to existing national ambitions for England as well as a generic health systems framework to illustrate gaps in existing recording tools and to iteratively develop a novel approach.

**Results:**

A combined total of 124 policy documents were identified and screened, in which 131 specific care home integration initiatives were identified. Current initiatives emphasized monitoring quality in care homes, workforce training, and service delivery changes (such as multi-disciplinary teams). There was comparatively little emphasis on financing or other incentive changes to stimulate provider behavior for the care home setting. We present a novel typology for capturing and comparing care home integration policy initiatives, largely conceptualizing which part of the system or specific transition point the care home integration is targeting, or whether there is a broader cross-cutting system intervention being enacted, such as digital or financial interventions.

**Conclusions:**

Our typology builds on the gaps in current frameworks, including previous lack of specificity to care homes and lack of adaptability to new and evolving initiatives internationally. It could provide a useful tool for policymakers to identify gaps in the implementation of initiatives within their own areas, while also allowing researchers to evaluate what works most effectively and efficiently in future research based on a comprehensive policy map.

## 1. Introduction

Health and social care systems in many countries have begun to trial and adopt so-called “integrated” approaches. The COVID-19 pandemic has emphasized the need for better integration within health and social care systems, especially in care home settings (1, 2). Care homes, in the UK, provide residential, and sometimes also nursing, care to mostly older adults or other vulnerable people who cannot be accommodated at home or in other settings (3). They can be run and owned by local government, or privately (4). Care homes provide significant bed capacity to particularly vulnerable and frail people, are frequently involved in hospital care transitions, and can influence health and economic outcomes, including substantial preventable care costs. As such, care homes are critical actors in the wider effort to integrate health and social care delivery.

The integrated care agenda builds on the global movement toward a person-centered approach to health and care delivery (5), “in which individuals, families and communities are served by and are able to participate in trusted health systems that respond to their needs in humane and holistic ways” (6). Therefore, integrated services aim to bring together previously fragmented services into a single “wrap-around” and coordinated pathway to better address patients holistic care needs. In this paper, integrated health and care services are defined as those “that are managed and delivered in a way that ensures people receive a continuum of health promotion, disease prevention, diagnosis, treatment, disease management, rehabilitation and palliative care services, at the different levels and sites of care within the health system, and according to their needs throughout their life course” (7).

Although this is a relatively broad definition of integration—which is recognized as a nebulous concept (8)—it captures the multiplicity of activities and interconnected factors, which can be implemented across the health system, that aim at effective health and care integration (9). In practice, interventions that attempt to accomplish these aims can take many forms. Some are directly focused on service delivery, such as multi-disciplinary team assessments and care planning. Others are more focused on back-office system change, such as up-skilling the workforce, encouraging increased communication *via* shared IT platforms, or changing incentive systems to try and encourage more collaborative behavior.

To date, evidence of effective integration surrounding care homes is limited, both in the United Kingdom and internationally (10), although some specific types of care transition interventions show promise for reducing readmissions to hospital (9–12). Looking at England specifically, current evidence of care home integration can be primarily identified from the Vanguard “New Care Models” programme implemented between 2015 and 2018 (13), and more generally for integration (not care home specific) from previous piloting approaches (14). The effectiveness of the overall Vanguard programme, including both care home and non-care home specific models, was mixed. Yet, models that focused specifically on care homes showed the most promise in slowing the rise in emergency hospital admissions, which was their primary aim (8). However, the Vanguards were selected as exemplary sites, provided with additional funding, and were not necessarily representative of wider approaches across the sector (15). Furthermore, individual pilot programmes were highly heterogeneous, and the programme evaluation was not able to identify which specific integration activities were enacted, let alone those that were key drivers of improvement or were (cost-)effective (8, 16). This more detailed analysis requires a more complete “policy map” as a first step, details of relevant interventions introduced by locality and year.

There have, however, been attempts focused specifically on capturing this more concrete nature of integration activity in care homes (1). A small survey study by Gage et al. (17) suggested that efforts to promote more effective collaboration among care home providers and local health services had primarily occurred “at the level of individual working relationships,” indicating a lack of formal service level or inter-organizational forms of integration among these sectors. More generally, there was reported to be a “lack of understanding between care homes and the National Health Service about how the two sectors should work together” to advance care integration (17). Further, a qualitative scoping report conducted by Baylis and Perks-Baker (18) examined how staff in care homes, health services, local authorities and CCGs experienced the process of collaborating more closely. From this, initiatives were identified that contributed to: improvements in care quality and continuity; residents' quality of life; resident involvement in care planning and review; prescribing practice; workforce outcomes; and documenting and symptoms recognition. Moreover, a recent literature review included in Lloyd et al. (19) identified evidence of initiatives that appear to drive care home improvement, especially when initiatives were used in combination. Such initiatives included: multi-disciplinary teams; partnership working between care home staff, GPs and other healthcare professionals; additional training for care home staff; the use of data for monitoring residents' outcomes; better preventative assessment and care management; advance care planning; end-of-life care planning; and medicines management. Nevertheless, the literature to date has been mostly based on surveys with low response rates, qualitative interviews with participants spanning a small number of areas, or non-systematic literature reviews based on the published evidence for “what works” rather than “what is enacted” more generally.

In addition, the variability in intervention reporting in the current literature highlights the lack of appropriate guidance for systematically recording and classifying interventions relevant to care home integration, i.e., a suitable framework for recording these concrete interventions. Many general integrated care frameworks exist but tend to differentiate at a more conceptual level (20), for example between horizontal (between services within a sector) or vertical (between different sectors) integration, rather than concentrating on classifying concrete examples of implementation. Many also encompass intangibles or latent variables, for example a focus on “culture” or “history,” which are extremely difficult to measure and even more difficult to translate to other settings. It is debatable how useful these frameworks can be for recording, comparing, and evaluating effectiveness of enacted interventions within and across areas.

There are also other specific integration frameworks, for example, related to multi-morbid patients and primary care-based activity, which could be more useful for mapping concrete examples of integration activity within those settings (21, 22). However, such flexible frameworks are currently not specific to our setting of interest, care homes. These more suitable mapping frameworks do, though, tend to build on generic health system building blocks (23), which describe the parts of the health system where changes can occur (e.g., “service delivery,” “financing”) (24). There are also more normative setting-specific frameworks, such as the NHS England Enhanced Health in Care Homes (EHCH) framework (25), related directly to the ambitions of the health and care integration Vanguards discussed above. Both generic health systems and normative frameworks might offer starting points for the mapping process, although it is not clear how suitable they are for this more flexible policy mapping aim.

While the research discussed above offers a strong starting point in relation to specific, practical examples of integrated care interventions, the existing evidence base does not enable systematic mapping of integrated care policies across local areas or on a national scale (26). This is significant in terms of policy and practice, given that better understanding of potential differential impacts of integrated care initiatives is crucial to measuring their effects on key outcomes, which can potentially lead to improved care quality in care homes. Such a framework is necessary to begin to understand what forms of integration work best and why, while also aiding the identification of key areas for improvement and encouragement of good practice both nationally and internationally (27).

### 1.1. Aim

The primary aim of this paper was to present a novel, general typology for mapping integrated care policies in the care home sector.

We fulfilled this by:

(i) Inductively, conducting an exemplary policy mapping exercise, utilizing the document analysis method, to identify and compare the types and extent of locally implemented integrated care policies related to care homes. Specifically, we classified qualitative data from care home-relevant policy documents in a devolved region in England.
(ii) Deductively, mapping this data to a standard health systems framework (24, 28), and to the normative national ambitions for England articulated in the Enhanced Health in Care Homes (EHCH) Framework (25) to assess the potential shortcomings of existing resources.

## 2. Materials and methods

### 2.1. Study design and setting

The initial policy mapping was conducted in a devolved region of England. The Greater Manchester (GM) city-region was selected as it offered a clearly defined geography and governance space large enough to provide sufficient data for the purposes of our study. GM encompasses 10 localities, with nearly three million residents. Collectively, it is governed by a Mayoral Combined Authority, and, in partnership with local NHS and social care partners, the city-region has devolved responsibility for its £6.6 billion health and social care budget (29). The pioneering series of devolution deals, agreed with the UK government, has enabled the city-region to use this autonomy to develop a comprehensive population health system approach (30). In terms of care homes specifically, GM has roughly 400, providing around 18,000 beds (exact numbers fluctuate over time as providers enter and exit the market) (31). Some localities, like Salford, have previously introduced novel interventions such as a virtual GP practice run by the local hospital to cover care home residents (32). The GM site, therefore, provides data on “real world” policy implementation, at the cutting edge of integrated care, but outside of the national EHCH Vanguard pilots and with policy variation within the geography.

We first undertook a qualitative, systematic policy mapping exercise of integration policies which specifically related to the care home sector across GM's 10 CCG localities. To do this, we conducted a systematic documentary search on a variety of official policy or strategy documents (see Section 2.2) to identify and extract a range of information on enacted integrated health and social care initiatives.

Document analysis has been described as “a systematic procedure for reviewing or evaluating documents—both printed and electronic material,” which enables data to “be examined and interpreted in order to elicit meaning, gain understanding, and develop empirical knowledge” (33). The document analysis method allows researchers to conduct in-depth analysis of the content of specific types of policy initiatives, including health policy (34). Documentary sources relating to health and social care policies are available in a plethora of forms and are written for statutory purposes or to address a wide range of policy matters. Examples of such materials include: official policy or strategy documents, implementation documents, legal documentation, and internal working documents (35). The outcome of document analysis is the extraction of data, usually in the form of “excerpts, quotations, or entire passages” (36) of text. Extracted data are typically organized into categories, major themes or case examples through content analysis (33).

When conducting the present document analysis, the research team adopted elements of the READ approach (Ready materials, Extract data, Analyze data, Distill; see below for details at each stage); a “systematic procedure for collecting documents and gaining information from them in the context of health policy studies at any level (global, national, local, etc.)” (35).

### 2.2. Readying source material

Our collection strategy focused primarily on the 10 Greater Manchester Clinical Commissioning Groups (CCGs). The CCGs were the primary focus given our predominant interest in the integration of care homes with healthcare, the latter of which CCGs are responsible for commissioning. We additionally supplemented data with relevant documentary sources from the 10 GM local authorities, responsible for social care and other public services, which have coterminous boundaries with the CCGs. Such organizations exercise significant policy and statutory responsibilities (e.g., commissioning, service delivery, and oversight) in relation to care homes. In fulfilling their responsibilities, CCGs and local authorities produce a number of policy documents, along with other information essential to care home policy.

A comprehensive approach to sourcing and extracting documents was adopted, which involved the identification of publicly available documents that appeared of potential relevance. We first sourced recently published and publicly available electronic versions of policy documents from GM CCG websites. Document searches were conducted during the period of January–April 2021. The most recently published versions of documents were sourced to ensure that the most up-to-date iteration of each organizations' policies were extracted.

Locality plans were also collected—documents that are co-produced by local authorities, CCGs, and other partners—which provided additional data on care home integration from a wider group of stakeholders. It is important to note that the range and type of available documents varied in each GM locality. While there were a number of common statutory documents (e.g., Annual Reports), some CCGs produced, or co-produced, additional locally focused documents, including care home specific documentation (see Table 1).

**Table 1:** Examples of Greater Manchester policy documents sourced from CCG and local authority websites.

Annual reports and accounts	Strategy for primary care
Delivery plan	Older people's strategy
Operational plan	Annual engagement report
Strategic plan	Communication strategy
Constitution document	Commissioning intentions
Mental health investment report	Corporate performance
Quality strategy	Sustainability report
Safeguarding strategy	Care home strategy
End of life care strategy	Primary care workforce development strategy
Stakeholder survey findings	Minutes and reports from committees

Once data saturation from official CCG and local authority documentation was reached, this formal collection of documents was supplemented with generic online searches to identify any additional relevant policy documents. Such searches included the websites of relevant local organizations, such as Health Watch, GM local authorities and local support groups (e.g., dementia support groups), as well as national bodies, such as Care Quality Commission inspection reports of care homes in the GM localities, and finally, general searches using keywords (on the Google search engine, e.g., “care home” + “locality name”).

### 2.3. Data extraction

Initially, a “broad brush” approach was adopted, to enable the extraction of all potentially relevant data from the documentary sources, using the generic subject fields of “integrated care” and “care homes” (including “residential and nursing homes”). The data extraction process involved thoroughly reading each of the sourced GM policy documents in turn, then conducting basic keyword searches of the documents using the generic subject field terms to reduce human error in the manual process. Finally, excerpts of text relevant to the research focus were extracted. Extracted excerpts were then recorded and categorized in a Microsoft Excel spreadsheet (see Supplementary material).

It was evident during the initial extraction process that there was a hierarchical structure to the data, which thus needed to be reflected in the coding and subsequent analysis. Accordingly, a “data categorization hierarchy framework” was developed (see Supplementary material), which permitted the categorization of data in order of relevance in relation to the study's research focus. In practice, data extraction iteratively focused on data surrounding recently developed integrated care strategies, policies and initiatives specifically designed for, or targeted at, the care home sector, including care home providers, staff, residents, and residents' family members or wider support group. Data specifically related to integrated care and care homes became the exclusive focus of the analysis thereafter. Since most document sources were produced by the healthcare sector, separate from the care home sector, we made the assumption that instances where the documents were outlining care home interventions were describing examples of integration.

### 2.4. Analysis

From the extracted excerpts, we inductively developed initial thematic categories to support interpretation of the data. Such categories focused on isolating three dimensions of care home related integration:

1) Aims: The overarching clinical areas or broad themes which are priorities for action detailed in the documents (e.g., improved dementia care for care home residents).
2) Activity: The initiative, policy, or intervention that is detailed as being implemented to address the key aims.
3) Provider(s): Integration with whom and how this is implemented in practice, where recorded.

We first described the documents and extracted data thematically across the three dimensions to contextualize the data prior to the policy mapping and framework analysis. Following this, only those extracts classified as reflecting an “activity” were analyzed in detail, since categorizing and mapping these activities was our primary aim.

In the second stage of coding, policy categories were assigned to each extracted excerpt. Such policy categories were initially derived deductively, based on the NHS England EHCH Framework's “elements” and “sub-elements” of care (25) and Atun et al.'s (24) and WHO (28) health systems framework (see Supplementary material). Mapping to Atun et al.'s (24) and WHO (28) framework allowed us to specifically describe which of the four dimensions of the health system (i.e., governance and organization; financing; resource management; service delivery) each activity was targeting. Mapping to the EHCH framework ensured that the extracted data were relevant to care home related integration and the wider NHS care home improvement agenda. As such, it also acted as a method of “quality checking” the data, which strengthened the overall validity of analysis. Further, mapping on to the EHCH framework allowed for the exploration of whether our data extraction had uncovered additional local “care elements/sub-elements” that may have been missed from the national EHCH Framework. We conducted descriptive analyses of the mapped categories to further describe and better understand the relevant policy initiatives.

Finally, as the data extraction and initial categorization progressed, an inductive, or data-driven, approach was adopted. The data-driven approach followed a continuous process of refining the policy categories as our understanding of the data evolved and deepened. After several phases of policy category refinement, involving discussion and exchanges of ideas among the research team, theoretical saturation was achieved where we struggled to further distill the categories we had constructed, and a final categorization typology was agreed by consensus.

## 3. Results

### 3.1. Document searches

**Figure 1:**
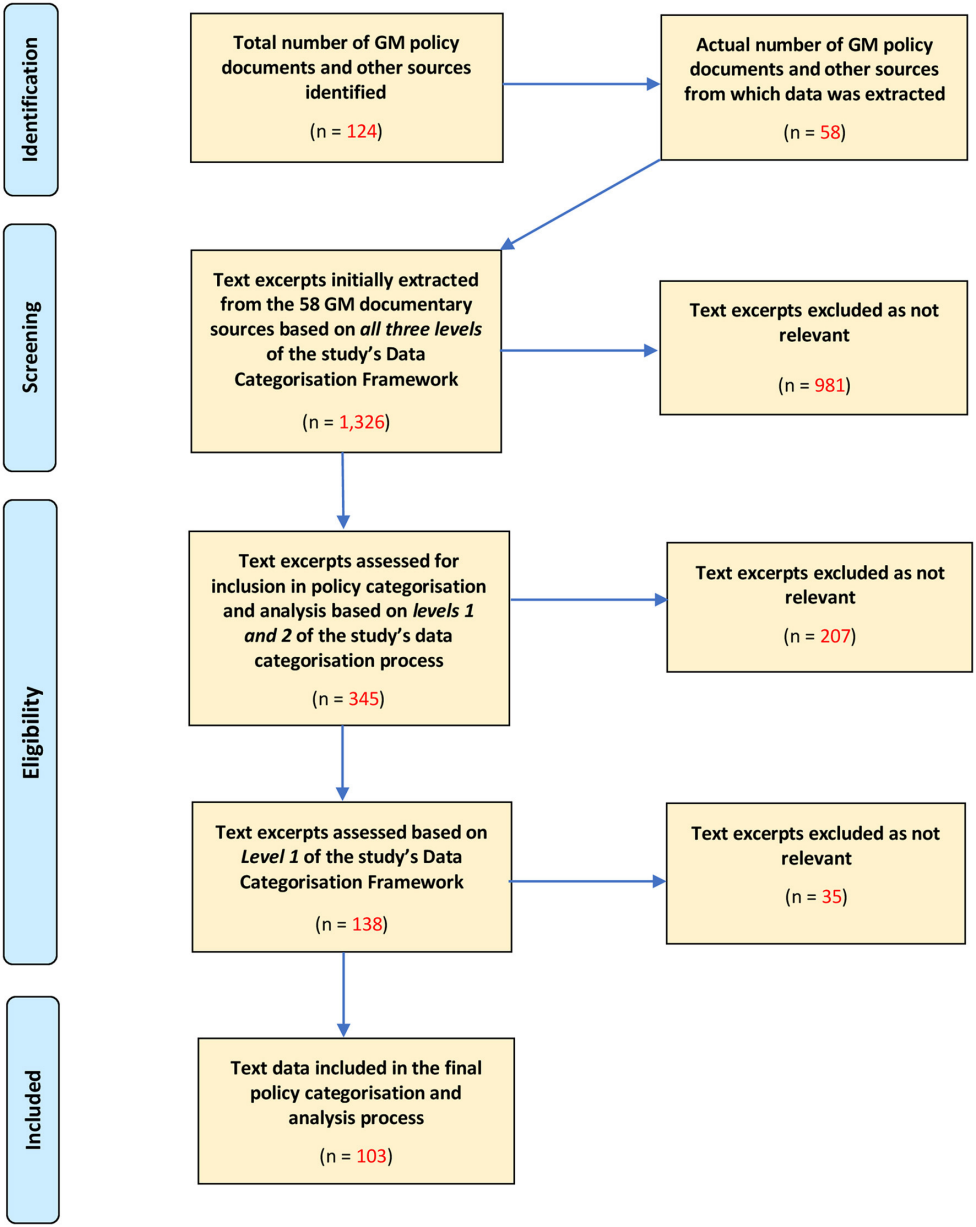
Modified PRISMA diagram explaining the study's data extraction and inclusion process.

The total number of documents identified from the 10 GM CCG websites was 97. Disaggregated across each of the 10 localities, the number of policy documents collected for each (shown in brackets) were: Bolton (9), Bury (10), Heywood, Middleton, and Rochdale (9), Manchester (8), Oldham (10), Salford (10), Stockport (10), Tameside and Glossop (8), Trafford (9), and Wigan (14). The supplementary searches produced a further 27 additional sources for eight of the 10 localities. A combined total of 124 documents were therefore identified and screened. A total of 58 of the screened documents were found to contain relevant data during the data extraction process (see Figure 1).

From the relevant documents 345 text excerpts were initially extracted as potentially relevant. Further rounds of screening, as described above, focusing primarily on those directly relevant to care homes and their residents, reduced the number of relevant text excerpts to 103, of which were included in the subsequent analysis.

### 3.2. Data description

A number of patterns emerged from the policy document analysis that are key to interpreting the policy mapping and framework results below.

First, the documents analyzed were mostly focused on GM CCG and local authority policy ambitions and aims toward the care home sector, rather than providing details of specific care home-related initiatives and processes by which these would be implemented (what we referred to as “actions,” above). Perhaps expectedly, it was also evident that there was a relative dearth of specific care home-related integration initiatives in many of the GM policy documents, including documents that were expected to be particularly relevant and significant, such as Strategic Plans, Operational Plans, and Annual Reports. More surprisingly, a similar phenomenon was observed in those documentary sources which were written with the explicit aim of detailing how care home related integration policies would be delivered and implemented in the GM localities. Nevertheless, we did manage to extract a number of relevant policies across all localities.

Policy development in relation to care home integration appeared to vary among the GM localities. It was evident, however, that some care home integration initiatives in GM were in response to national care home policy priorities, such as the Red Bag hospital transfer scheme, the React to Red pressure ulcer intervention, and implementation of the national EHCH model; all nationally driven agendas and common across many localities. However, it was also the case that some localities had developed specific local policies. In some cases, it appeared that certain localities were compelled to act to develop care home integration policies in response to previous poor performance ratings of care homes from care home inspections. Other areas of activity seemed to reflect specific local contexts which had been identified as priorities for action by policymakers and other actors in GM localities. Further, it was evident that some localities had developed a comparatively more comprehensive agenda for care home related integration. This was most evident in those localities which had developed dedicated care home strategies.

### 3.3. Mapping of findings to existing analytical frameworks

Focusing specifically on the “actions” (i.e., the specific care home integration initiatives), 131 unique “actions” were identified across the 10 GM localities, with multiple actions described in some text excerpts.

#### 3.3.1. Findings in relation to the wider health system

Many integrated care initiatives are known to span more than one domain of a health system, given the complexity of the interventions. For example, introduction of a multi-disciplinary team could be considered both a “Resource Management” change (workforce reconfiguration) as well as a “Service Delivery” change (as it also changes the way care is delivered to the patient). More than half (53%, *n* = 69) of the initiatives we identified fit into more than one health system domain, and 14% (*n* = 18) spanned three or more of Atun et al.'s (24) four health system domains.

Non-exclusively, 69% (*n* = 91) of the initiatives were related to Resource Management; many related to human resource management, such as workforce training. Around half (49%, *n* = 64) of initiatives were related to Governance and Organization, including oversight boards and quality monitoring. Forty-two percent (*n* = 55) of initiatives also had an element of Service Delivery change, including use of new multi-disciplinary teams, pharmacy support, and care planning. Relatively few initiatives (10%, *n* = 13) were linked to the Financing domain of health systems transformation, which included integration initiatives such as pooled budgets.

#### 3.3.2. Findings in relation to the EHCH framework

While the EHCH framework categories are much more specific than the wider health system domains, we identified that a third (33%, *n* = 43) of initiatives still spanned more than one of the EHCH domains. The spanning of initiatives across multiple EHCH categories is largely a result of the category labels being so specific that many interventions did not fit neatly into a single category, and so two or more categories were often assigned to attempt to more fully capture what the initiative involved.

In line with the mapping to the wider health system, the most frequent (27%, *n* = 36) EHCH category aligned with human resource management and up-skilling, specifically: “6.1 Training and development for social care provider staff” (usually by healthcare staff). Other frequent categories included: “5.1 Co-production with providers and networked care homes” (14%, *n* = 18), “7.3 Better use of technology in care homes” (12%, *n* = 16), “5.2 Shared contractual mechanisms to promote integration (including Continuing Healthcare)” (12%, *n* = 16), “6.2 Joint workforce planning across all sectors” (11%, *n* = 14), and “1.2 Medicine reviews” (8%, *n* = 11).

### 3.4. A new typology of care home integration policies

Learning from the data and attempting to address the classification limitations identified in the existing frameworks, we developed a thematic typology focused on classifying initiatives (i.e., practical “actions”) specific to care home integration (see Table 2).

**Table 2:** A typology of care home integration policies and specific initiatives.

**No**.	**Care home policy categories**	**Indicative examples of policy initiatives**
**Defined providers/transition points?**		
1.	“Admissions/transfers” (i.e., targeting the transitions between different services, in particular discharge and transfer to/from Care Home sector)	• Red bag scheme (where care home staff pack a dedicated Red Bag for a patient needing hospital care containing vital existing condition/medication information to ease information transfer across settings) • Reducing hospital admissions (using risk assessment and triage tools to attempt to reduce avoidable hospital admissions and emergency calls) • Pathfinder initiative (identify patients who can be treated in care homes rather than in A&E) • Night Sitting Service (providing support overnight to those patients who attend A&E)
2.	“Preventative/rehabilitative” (i.e., pre-/post-healthcare specific)	• “React to Red” protocol (wounds, pressure sores, and ulcers prevention/treatment interventions) • Falls prevention, frailty • Nutrition, malnutrition, and dehydration (incl. fluid balance monitoring) initiatives • Rabblement and rehabilitation • Oral care
3.	“Healthcare organization monitoring, assessment, and quality improvement” (i.e., governance and oversight—commissioning-level)	• Local metrics for care homes • Care home dashboard (indicators) • Care home market management and strategy • Monitoring outcomes/quality of care by CCG/LA • Care home support toolkit to support complaints process • Risk assessment (e.g., Risk matrix to enable the early identification of the Care Homes/Providers requiring clinical intervention and support) • Safeguarding • Quality and safety strategy • Care home strategy • Care home excellence initiatives
4.	“Medicines review and optimization” (i.e., pharmacy within the care home)	• Prescribing • Administering medication (insulin in care homes project) • Medications support to care homes • Ordering medication/proxy ordering • Homely remedies policy
5.	“Improved primary care and specialist care” (i.e., primary/secondary care within the care home, also MDTs here involving both potentially)	• Enhanced nursing services for care homes (including the Nursing Home Service) • Enhanced primary care services • Alignment of care homes to a single Primary Care Network (*PCN*) • Weekly Home Round/'Weekly Check-in'/Ward Round • MDT and Hub Support (e.g., creation of a dedicated hub to co-ordinate the out of hospital urgent care response, with a special emphasis on care homes)
6.	“Care home collaborative, liaison and support” (i.e., inter-care homes)	Dedicated/specialist care home personnel and groups/teams including: • Care Home Support Group • Care Home Liaison Service • Care Home Support team • Care Home and Domiciliary Care Reform Board
**Cross-cutting/system initiatives?**		
7.	“Workforce development/training” (i.e., care home workforce directly, or up-skilling other workforce to work with/in care homes)	• Enhanced care worker role • Care home career promotion schemes • Taking action to ensure training and development opportunities are available to private sector care home staff • Initiatives to identify specific and ongoing training needs of care home staff • Developing care homes as future learning environments • Developing organizational culture and values • Networking, peer support or buddy schemes for care home managers
8.	“Use of technology to deliver care” (i.e., the more practical and delivery aspect of digital health)	• Virtual health and care services/consultations • Technology use to address social isolation and loneliness
9.	“Development of records, information and data sharing” (i.e., digital innovations to improve data/records sharing and ease of access to data among service providers and clinical/care personnel)	• Digital shared access to records • Digital capacity tracking/electronic real time care home bed occupancy • Sharing and gathering intelligence/information • Shared care plans
10.	“Personalized care and care planning” (i.e., whole system co-ordination activity)	• Personalized care • Case management approaches • Care planning improvement • Urgent care
11.	“Pooled budgets or provider payment incentives” (i.e., aimed at wider system inventive changes, or specific P4P/other specific incentive payments to enhance performance/outcomes)	• Better Care Fund • Local pooled budget initiatives • Nationally determined Directed Enhanced Services (e.g., EHCH DES) • Locally determined Local Enhanced Services (e.g., Care Home LES)
12.	“Care home transformation funding” (i.e., aimed at creating additional financial resource)	• Dedicated new funding streams for care home integration and improvement

A&E, Accident and Emergency; CCG/LA, Clinical Commissioning Group/Local Authority; PCN, Primary Care Network; MDT, Multi-Disciplinary Team; P4P, Pay-for-performance; EHCH, Enhanced Health in Care Homes; DES, Directed Enhanced Service; LES, Local Enhanced Service.

The developed thematic typology differentiates between two main conceptual classes:

(i) “Defined providers/transition points?”—capturing those initiatives focused on integration with a specific other provider type (e.g., pharmacy) within the health and care system, or on specific transition points between these providers.
(ii) “Cross-cutting/system initiatives?”—alternatively capturing those broader initiatives, such as wider workforce up-skilling initiatives, care planning initiatives aiming to improve coordination across multiple services, technology changes to deliver services across settings and share information, or wider financing and payment changes, which aim to change the wider system.

Within each of the conceptual classes, there are six “Care home policy categories,” which detail (i) the common providers with whom care homes are integrated or specific transition points targeted, and (ii) the common system-wide changes observed in the data. Finally, in the right-hand column of the framework, there are more specific “Indicative examples of policy initiatives.” The specific examples include common labels used to describe similar initiatives across the localities. Such labels are flexible, unlike the EHCH fixed prescriptive labels, and so can evolve as additional policy initiatives are identified (for example, in other localities, nationally and internationally), and can also be altered in terms of the specificity of the labels to suit the individual research goals.

In our dataset, 69% (*n* = 91) of initiatives were classified under the conceptual class describing integration of care homes primarily with a single other provider type, or a specific transition point. Thirty-one percent (*n* = 40) were instead focused on wider cross-cutting system initiatives.

The most common initiatives were related to: 3. “Healthcare organization monitoring, assessment, and quality improvement” (24%, *n* = 31); 7. “Workforce development/training” (14%, *n* = 18); 5. “Improved primary care and specialist care” (12%, *n* = 16); 2. “Preventative/rehabilitative care” (11%, *n* = 14); and 1. “Admissions/transfers” (10%, *n* = 13).

Finally, an important differentiator we identified in the initiatives, but least well-described systematically, was related to the extent of policy roll-out. For example, some policies were specifically aimed at individual long-term conditions (e.g., dementia), or clinical areas (e.g., end-of-life care), whereas others appeared to be more generally targeted at care homes. Policies also varied in the extent that they focused on the population of care homes/residents; for example, targeting care home residents exclusively, or care home residents plus non-care home residents as part of a wider health and care system reform. Despite financing of care homes varying between public and private funding, there appeared to be very little differentiation in describing this important aspect in terms of interventions too. We further experienced difficulty identifying which specific care home integration initiatives had already been, or were in process of being, implemented due to ambiguous wording. When mapping policies for quantitative analysis purposes, the extent of the roll-out and the timing of initiative implementation would be important to capture.

## 4. Discussion

### 4.1. Summary of findings

Empirical analysis of publicly available policy documents in the GM city region in England highlighted the limitations of current policy reporting related to integrated care initiatives, but also that these documents do provide a starting point for capturing local variation in integrated care initiatives related to care homes. While there is a clear national direction of care home integration-related policy, there is also local innovation, which is important to capture. Current initiatives in GM showed an emphasis on monitoring quality in care homes, workforce training, and service delivery changes (such as MDTs). We found comparatively little emphasis on financing or other incentive changes to stimulate provider behavior for the specific care home setting.

Building on the limitations identified from mapping initiatives on to existing frameworks, we present a novel typology for capturing and comparing care home integration policy initiatives. This new typology conceptualizes which part of the system the care home is integrating with, or whether there is a broader cross-cutting system intervention being enacted. Currently, from the analysis of GM policy initiatives, the emphasis appears to be on the former, integration with a single other provider or at a particular transition point for care pathways. The framework also builds on previous work by providing additional flexibility to classify initiatives on a more specific level. The ability to classify initiatives more specifically is essential for policy mapping local variation and innovation, and for subsequent statistical analysis to determine (cost-)effective approaches.

### 4.2. Strengths and limitations

Document analysis proved to be an efficient and effective method of collating data surrounding health and social care integration policies. The documents were relatively easy to obtain, clearly structured, accessible, and manageable, and contained a range of rich descriptive policy data. The analysis of already available documents allowed for a less resource and labor-intensive research approach to be adopted compared with alternative research methods that require primary data collection, such as surveys. Moreover, document analysis involving publicly available documents is a method that is transferable to other regional geographies and at a national scale in England, as well as internationally, permitting the recording of what initiatives are being enacted and the evaluation of associations with outcomes of policy interest. While specific initiatives might vary by context, our typology could be flexibly used to incorporate these differences where necessary.

The document analysis method, however, also has a number of limitations. Although document analysis has been used relatively frequently in health research, there remains a lack of definitive guidance on how this method should be applied in the context of policy (35). For practical reasons, we here concentrated on the most recently available documents, where other previously implemented initiatives might be reported in documents in other years. However, as our purpose here was to create a typology, as long as the initiatives did not differ systematically over time the typology should be adaptable to other time periods. Secondary source documents are not designed for research use and extracting consistent, comparable and comprehensive data of sufficient quality can be a challenge. On their own, documents often provide relatively little information, and even in cases where useful data can be retrieved, there may still be critical gaps which can hamper analysis and interpretation. It is rarely possible to explore the deeper context and biases that lie beneath policy statements, such as the underlying motivations, assumptions or priorities informing the authors, or which actors had most influence in shaping and determining policy formulation and prioritization set out in documentary sources (33, 37). It is therefore important to relate and interpret documentary data within the wider context within which it is situated.

As Bowen (33) points out, “in an organizational context, the available (selected) documents are likely to be aligned with corporate policies and procedures and with the agenda of the organization's principles.” Consequently, the breadth of data retrieved was framed by the organizational context from which it emerged and as such reflects each organization's priorities, concerns, and biases, especially those of key policy and decision makers within each organization. It is therefore important to acknowledge this subjectivity inherent in documentary research to ensure the credibility of the research (37). In this regard, the research team recognized that the GM policy documents were primarily produced by personnel from NHS organizations and local authorities. Therefore, these sources are likely to reflect primarily healthcare and local government priorities, rather than those of actors in social care and care home sectors directly.

The fact that the sourced documents were mainly focused on outcomes they aimed to achieve, rather than implementation of practical activities, may also reflect the nature of the secondary data sources collected. Many of the documentary sources were higher level policy documents, the purpose of which was to articulate strategic goals and targets of CCGs and local authorities, rather than provide details of specific interventions which would be implemented. Documents specifically related to the latter were largely absent from the public domain. The lack of detail related specifically to care home policy may, therefore, indicate a lack of focus on or prioritization of care home-related integration, or more prosaically, it may simply have been an oversight on behalf of policymakers and key health and social care system leaders. Our primary focus on extracts explicitly related to care homes/residents also means we might have missed some broader interventions affecting the whole health and care system (but not specific to our setting of interest). This might be especially pertinent to financial interventions, such as pooled budgets, for instance.

Finally, the nature of the data analyzed prevented us from capturing potentially important pre-conditions for integration, such as historical working relationships and broader organizational culture. However, such pre-conditions are also arguably least transferrable to other contexts, so might not be the most meaningful aspects to capture in a policy map.

### 4.3. Interpretation of findings

Compared to other dimensions within the wider health and social care integration agenda, the findings of the present study suggest that specific care home related integration policy work is at a relatively early stage of development, at least within the GM region. However, there were indicators inherent in some of the more recently published documentary sources that the COVID-19 pandemic has led to an increased focus on the care home agenda among policy makers. As a consequence, the impact of the pandemic may well-accelerate the implementation of care home related integration initiatives.

In comparison to previous attempts to map care home integration, our findings suggest that there is an increasing focus on formal service level or inter-organizational integration. Importantly, this finding reflects a notable change since Gage et al.'s (17) survey conducted in 2012, where a lack of formal service level and inter-organizational integrated approaches was evidenced. However, our findings also suggest that such approaches are mostly still focused on pathways with single other services rather than cross-system or large organizational incentive changes. Such findings perhaps indicate a marginal move toward a more fully integrated system, but that the majority of changes remain smaller-scale and more conservative than revolutionary.

In addition, we identified similar integrated care initiatives to those described in previous research, such as multi-disciplinary teams, medication management and care planning (18, 19). Our findings build on previous research findings by mapping policy initiatives more systematically using an innovative, specially developed typology, and by showing that this mapping appears to be achievable using publicly accessible documents rather than more labor-intensive surveys or academic literature review approaches.

Our mapping of an entire devolved health and care system, and comparison to the national approach, exemplified by the EHCH programme, also highlights important local variation in policy and potentially important drivers of change. For example, the regulator rating could be a possible driver of a focus on quality improvement at the commissioning level (an initiative itself not easily mappable using the EHCH framework), and for additional training of staff across the health and social care system to deliver improved care.

### 4.4. Implications for policy and research

Our proposed typology may provide a “menu” of possible policy choices for policymakers, and/or, a structure to map their own initiatives and provide systematic comparisons for quantitative and qualitative evaluations for researchers. When we presented the typology to individuals from the clinical population and the general population, they also informed us that the typology was useful for describing integrated care to the general public in a more concrete way.

Critically, there was only limited reference in the documentary sources to evaluation having been conducted to assess the efficacy of care home related initiatives. However, Wigan's “Hospice in Your Care Home” project (38) and the Red Bag Scheme Hospital Transfer Pathway in Tameside and Glossop were in fact subject to evaluation (39). Given the large variation in approaches, it is important to evaluate what works, for whom, and in what context, in order for limited resources to be used most efficiently.

Local reporting and evaluation of initiatives will also be important for monitoring the impact of the most recent implementation of the EHCH framework, *via* incentivization of Primary Care Networks and groups of primary care practices. As noted above, the EHCH framework provides a prescriptive set of nationally decided initiatives. It is not clear that the same set of initiatives would be optimal for all localities across the country, or whether a focus on these initiatives will disrupt local innovation responses to locally identified problems.

Future research could use our reported typology to more fully map care home integration activity over geography and time so as to be able to examine the relative (cost-)effectiveness of various concrete approaches. This outcome evaluation, by population sub-group, could also be used to document and address potential inequities in provision. Different approaches pre- and post-COVID-19 are also likely to be substantial and important to capture effectiveness of. At the full mapping stage it would also be useful to have validation from key informants from the organizations who were leading or coordinating care home focused work.

## 5. Conclusions

Through the document analysis method, CCG and local authority documentary sources were revealed to provide a good starting point for mapping care home integration policies, although their detail could be improved further in future iterations. Current policies related to integrated care in care homes appear to be at an early stage, smaller scale, focused on integration with single providers, or on more general quality monitoring by healthcare agencies Current frameworks are not suitable for fully capturing the variation in integrated care initiatives. We thus present a novel typology for classifying integrated care initiatives which builds on the gaps in current frameworks and should provide a useful tool for policymakers, while also allowing researchers to evaluate what works most effectively and efficiently.

## Data availability statement

The original contributions presented in the study are included in the article/Supplementary material, further inquiries can be directed to the corresponding author.

## Author contributions

JS and MM conceptualized the study. GS, CE, and JS contributed to the initial study design. GS and CE collected the data and conducted the analysis. GS wrote the first draft of the manuscript. All authors contributed to the final study design, reviewed the methodology and results, and provided critical revisions to the final submitted manuscript.
