# Supplementary material for: A typology of integrated care policies in the care home sector: A policy document analysis
Source: Front Public Health. 2023 Feb 21;11:943351. doi: 10.3389/fpubh.2023.943351 (PMC9989008; doi:10.3389/fpubh.2023.943351)
Supplement: Supplementary file 1 [file Table_1.DOCX]

Supplementary Material

# Data extraction spreadsheet

**Supplementary Table 1.** Data extraction sheet example

# Policy Level Hierarchy

**Supplementary Table 2.** Policy level hierarchy development

| **Data relevance & weighting** | **Description** |
| --- | --- |
| **Level 1**  **(focus of this research)** | Recently developed integrated care strategies, policies and particular initiatives *specifically* designed for, or targeted at, the care home sector, including care home providers, staff, residents, and residents’ family members or wider support group. |
| **Level 2** | Recently developed integrated care strategies, policies and specific initiatives focused on or targeted towards the older adult cohort more generally, not only those receiving care in care homes. |
| **Level 3** | Recently developed strategies, policies and specific initiatives designed to support wider dimensions of health and social care integration, which may have some relevance to the care home sector. |

# Existing framework categories

**Supplementary Table 3.** Atun et al.(1) Health systems framework

| 1. Governance and organisation | "(i) governance and organization: the policy and regulatory environment, stewardship function of the ministry of health and relation of the MoH with other levels of the health system, and structural arrangements for insurers/purchasers, healthcare providers and market regulators" |
| --- | --- |
| 2. Financing | "(ii) financing: how the funds are collected, funds and risks pooled, finances allocated within the health system and how healthcare providers are remunerated" |
| 3. Resource management | "(iii) resource management: how resources – physical, human and intellectual – are generated and allocated, including their geographic and needs-based allocation" |
| 4. Service delivery | "(iv) service delivery: includes both population and individual level public health interventions and healthcare services provided within the community, PHC, hospitals, and other health institutions" |

**Supplementary Table 4.** NHS England Enhanced Health in Care Homes (EHCH)(2) framework

| 1. Enhanced primary care support | 1.1 Each care home aligned to a named PCN, which leads a weekly multidisciplinary ‘home round’ |
| --- | --- |
|  | 1.2 Medicine reviews |
|  | 1.3 Hydration and nutrition support |
|  | 1.4 Oral health care |
|  | 1.5 Access to out-of-hours/urgent care when needed |
| 2. Multi-disciplinary team (MDT) support including coordinated health and social care | 2.1 Expert advice and care for those with the most complex needs |
|  | 2.2 Continence promotion and management |
|  | 2.3 Flu prevention and management |
|  | 2.4 Wound care – leg and foot ulcers |
|  | 2.5 Helping professionals, carers, and individuals with needs navigate the health and care system |
| 3. Falls prevention, Reablement, and rehabilitation including strength and balance | 3.1 Rehabilitation/reablement services |
|  | 3.2 Falls, strength, and balance |
|  | 3.3 Developing community assets to support resilience and independence |
| 4. High quality palliative and end-of-life care, Mental health, and dementia care | 4.1 Palliative and end-of-life care |
|  | 4.2 Mental health care |
|  | 4.3 Dementia care |
| 5. Joined-up commissioning and collaboration between health and social care | 5.1 Co-production with providers and networked care homes |
|  | 5.2 Shared contractual mechanisms to promote integration (including Continuing Healthcare) |
|  | 5.3 Access to appropriate housing options |
| 6. Workforce development | 6.1 Training and development for social care provider staff |
|  | 6.2 Joint workforce planning across all sectors |
| 7. Data, IT and technology | 7.1 Linked health and social care data sets |
|  | 7.2 Access to the care record and secure email |
|  | 7.3 Better use of technology in care homes |

# References

1. Atun R, Aydin S, Chakraborty S, Sümer S, Aran M, Gürol I, et al. Universal health coverage in Turkey: enhancement of equity. The Lancet. 2013;382(9886):65-99.

2. NHS England, NHS Improvement. The Framework for Enhanced Health in Care Homes 2020 [Available from: <https://www.england.nhs.uk/wp-content/uploads/2020/03/the-framework-for-enhanced-health-in-care-homes-v2-0.pdf>.
